# Extracellular vesicles derived from macrophages: Current applications and prospects in tumors

**DOI:** 10.3389/fbioe.2022.1097074

**Published:** 2022-12-15

**Authors:** Kecheng Lou, Shangzhi Feng, Hui Luo, Junrong Zou, Guoxi Zhang, Xiaofeng Zou

**Affiliations:** ^1^ The First Clinical College, Gannan Medical University, Ganzhou, Jiangxi, China; ^2^ Department of Urology, The First Affiliated Hospital of Gannan Medical University, Ganzhou, Jiangxi, China; ^3^ Institute of Urology, The First Affiliated Hospital of Gannan Medical University, Ganzhou, Jiangxi, China; ^4^ Jiangxi Engineering Technology Research Center of Calculi Prevention, Ganzhou, Jiangxi, China

**Keywords:** extracellular vesicles, macrophages, nanomedicine, engineered extracellular vesicles, tumor vaccines

## Abstract

Macrophages (Mφs) are significant innate immune cells that perform a variety of tasks in response to different pathogens or stimuli. They are widely engaged in the pathological processes of various diseases and can contribute to tumorigenesis, progression and metastasis by regulating the tumor microenvironment and cancer cells. They are also the basis of chemoresistance. In turn, the tumor microenvironment and the metabolism of cancer cells can limit the differentiation, polarization, mobilization and the ability of Mφs to initiate an effective anti-tumor response. Extracellular vesicles (EVs) are small vesicles released by live cells that serve as crucial mediators of intercellular cell communication as well as a potential promising drug carrier. A growing number of studies have demonstrated that Mφs-EVs are not only important mediators in the pathological processes of various diseases such as inflammatory disorders, fibrosis and cancer, but also show significant potential in immunological modulation, cancer therapy, infectious defense and tissue repair. These natural nanoparticles (NPs) derived from Mφs are believed to be pleiotropic, stable, biocompatible and low immunogenic, providing novel alternatives for cancer treatment. This review provides an update on the pathological and therapeutic roles of Mφs-EVs in cancer, as well as their potential clinical applications and prospects.

## Introduction

Despite the increasing efficacy of anti-cancer therapies, global cancer death rates continue to rise (Maeda and Khatami, 2018). It is estimated that about 10 million cancer deaths will occur in 2020, and this figure is likely to rise further due to increased risk factors associated with globalization and economic growth (Sung et al., 2021). Currently, cancer therapies include surgical intervention, radiation therapy and the administration of chemotherapy medications, but this typically kills healthy cells and causes toxic reactions in patients. Furthermore, cancer persistence is a critical obstacle to cure cancer, and factors such as tumor recurrence, improper physicochemical features of anticancer drugs and the associated poor pharmacokinetic profile often influence the fate of treatment and lead to reduced efficacy (Minchinton and Tannock, 2006). Among these limitations, multiple drug resistance (MDR) is one of the main factors contributing to the lack of efficacy, which is obtained by lowering cancer’s responsiveness to standard chemotherapeutic drugs as a result of efflux pump overexpression (Giddings et al., 2021). The hypoxic condition of the tumor microenvironment (TME) (Robey et al., 2005), the modification of epigenetics (Assaraf et al., 2019) and the influence of cancer stem cells (Zhou et al., 2021). With the maturation of nanotechnology, several new therapeutic methods for cancer are progressively being available to the public (Zaimy et al., 2017; Dadwal et al., 2018; Jeswani et al., 2018; Wang et al., 2021a).

Mφs are essential innate immune cells in the body that found in almost all tissues and contribute to immunity, repair and homeostasis *in vivo* (Gentek et al., 2014). They evolved from extremely heterogeneous hematopoietic progenitor cells to execute multiple activities that are dependent on various triggers. Mφs are the first line of defense against pathogen invasion and exhibit different phenotypes in response to a variety of endogenous and exogenous danger signals that mediate immune system and tissue homeostasis. Tumor-associated immune cells and TME have a deeply influence on tumor progression (Anderson and Simon, 2020), while Mφs play a crucial role in tumor suppression or progression.

EVs are membrane-enclosed vesicles that are naturally released by almost all cell types. EVs are classified into multiple subtypes based on their different origins and sizes, and are an important information carrier capable of transferring cargo from the parent cell to the recipient cell, regulating the physiological or pathological processes of the recipient cell, with cargo packed with cellular proteins, nucleic acids and metabolites. Moreover, EVs can mediate autocrine, paracrine, and endocrine processes (Andaloussi et al., 2013; Xu et al., 2013; Record et al., 2014), and are regarded as a useful diagnostic and therapeutic tool for illnesses.

With the investigation on the functions of Mφs-EVs in diverse disease states, there is increasing evidence that Mφs-EVs play a critical role in the disease progression (Wang et al., 2020; Wang et al., 2021b; Feng et al., 2022). They can facilitate cell-cell and organ-cell communication in cancers by delivering nucleic acids and proteins (Cianciaruso et al., 2019; Wang et al., 2020; Pu et al., 2021; Barone et al., 2022), and are key factors affecting TME. Thus, a comprehensive understanding of Mφs-EVs and their roles in disease pathogenesis as well as in therapy will provide us with novel ideas for tumor treatment in the future.

### Macrophages (Mφs)

Mφs in tissues and monocytes in peripheral blood are now commonly classified as mononuclear phagocytic system (MPS), with Mφs originating from bone marrow stem cells. In addition to this, Mφs may have at least four sources: F4/80^hi^ Mφs from the yolk sac, F4/80^lo^ Mφs from the bone marrow, Langerhans cells from the fetal liver, and extramedullary hematopoiesis (Cortez-Retamozo et al., 2012; Satpathy et al., 2012; Wynn et al., 2013; Shand et al., 2014; DeNardo and Ruffell, 2019). Mφs are broadly divided into two types: tissue residing Mφs and infiltrating Mφs. Tissue residing Mφs include hepatic Kupffer cells, microglia, peritoneal, lung, splenic red pulp, and bone marrow (BM) Mφs, which are defined as residing in their respective tissues in a stable state and performing homeostatic activities (Murray and Wynn, 2011). Infiltrating Mφs, on the other hand, are discovered only after an inducible lesion (Hashimoto et al., 2013). It is generally documented that classical monocytes (also known as “inflammatory” or Ly6Chigh monocytes) are the source of infiltrating Mφs found in pathological conditions, such as cancer, atherosclerosis, and metabolic diseases (Robb et al., 1995; Sakagami et al., 2009; Qian et al., 2011).

The phagocytic activity of Mφs eliminates dead and dying cells, and in turn, the elimination of cellular debris provides Mφs with nutrients. Even though Mφs are plentiful in TME and can activate T cells to defend against tumors, they frequently fail to do so as weak antigen presenters against developing tumors, resulting to immunosuppression (Broz et al., 2014; Muraoka et al., 2019). In addition, signaling signals in TME alter Mφs metabolism, which has a broad impact on their biology and function that extends far beyond antigen presentation. Tumors reprogram Mφs metabolism to limit Mφs-mediated inflammation and tumor cell death.

Mφs are highly diverse immune cells that respond to a wide range of stimuli. Mφs exhibit variable phenotypes depending on their microenvironment and can be broadly categorized into two subtypes: classically activated Mφs (CAMs, M1 Mφs) and alternatively activated Mφs (AAMs, M2 Mφs) (Mackaness, 1962; Mehla and Singh, 2019; Liu et al., 2020). In general, M1 Mφs predominate in early inflammation against danger signals and can demonstrate a strong pro-inflammatory response before shifting to M2 Mφs, which then play an immunomodulatory role, supporting tissue repair, regeneration, and fibrosis rather than acting as effective “killers” (Reiner, 2009). In tumors, altered Mφs metabolism promotes not only direct Mφs function but also changed the transcriptional activation status of functionally important cytokines. Tumor-associated Mφs (TAMs), the most prevalent immune cell population in tumors (Noy and Pollard, 2014), are predominantly derived from circulating monocytes and serve as immunosuppressive location that promotes tumor invasion. TAM antigen presentation is a critical part linked with immune resistance, and TAM status and function have a significant impact on tumor immunosensitivity. In fact, TAMs are more likely to be M2-type and promote tumor growth by causing immunosuppression (Peterson et al., 2016; Fu et al., 2020; Yang et al., 2021). However, since tumors are evolving tissues and molecules within the TME and change at different phases, the phenotype of TAMs is dynamically altered in response to different TMEs (Komohara et al., 2016; Chen et al., 2019). TAM depletion or reprogramming would be an effective way to improve the efficacy of immunotherapy (Zeisberger et al., 2006; Muraoka et al., 2019).

Various approaches have been proposed in this context to reduce or modify TAMs in TME, including drug delivery systems, engineered EVs, and tumor vaccines, and drug delivery systems, in particular, have been widely used to achieve this goal due to their appropriate diversity in terms of delivered bioactive and surface-modified structures (Figueiredo et al., 2021; Peng et al., 2021; Hou et al., 2022). Moreover, with the growing interest in Mφs-EVs, there is increasing evidence that it can be exploited as a tumor marker as well as to enable individualized targeted nanomedicines and tumor vaccines, thus manipulating the anticancer effects of TAMs (Guo et al., 2020; Guo et al., 2022; Wei et al., 2022). Therefore, the application of Mφs-EVs in tumors will be an unstoppable trend, and a comprehensive understanding of Mφs-EVs and their role in disease pathology and therapy is needed to build the framework for a better future role for Mφs-EVs.

### Extracellular vesicles (EVs)

Since their discovery more than 3 decades ago, EVs are a heterogeneous set of membrane structures containing intracellular molecules with a wide range of physical properties and biological origins, a new mode of cellular communication, which can be divided into two categories basing on the size, one is vesicles of 50–1000 nm formed by the germination of the plasma membrane, mainly including microvesicles (i.e., shedding vesicles, microparticles and microvesicles) and apoptotic vesicles; the other category is exosomes from the somatic membrane with a size of 40–160 nm (Kubo, 2018). EVs are of abundant origin and are widely prevalent in biological fluids (Taylor and Gerçel-Taylor, 2005; Admyre et al., 2007; Runz et al., 2007; Chen et al., 2010; Fernández-Llama et al., 2010; Reinhardt et al., 2012; Street et al., 2012; Levänen et al., 2013), and all currently known live cells can release EVs enriched with RNA, bioactive lipids and proteins (Couzin, 2005), all of which have a wide spectrum of biological roles. They participate in pathological and physiological regulation processes by delivering these substances within the organism. Table 1 lists the advantages and disadvantages of each EVs isolation technique.

**TABLE 1:** Isolation and purification method of major EVs.

Technique	Principle	Advantages	Disadvantages	Reference
Differential centrifugation	Different sizes and densities of particles have different settling rates during ultracentrifugation	• Ease operation	• Time consuming	Monguió-Tortajada et al. (2019); Gutiérrez García et al. (2020)
		• High purity	• Low recovery	
		• Suitable for large volume preparation	• High equipment cost	
			• Possible structure damage	
Size-exclusion chromatography	EVs are eluted by a porous stationary phase	• Maintain the native state of EVs	• Low yield	Foers et al. (2018); Davis et al. (2019)
		• High purity		
Tangential flow filtration (TFF)	EVs pass through a membrane with defined pore size or molecular weight cut-off	• Fast isolation process	• Vesicle clogging and trapping	Zeng et al. (2022)
		• Low equipment cost	• Unspecific method	
		• Large scale isolation		
Immunoaffinity	Specific binding between surface marker proteins and immobilized antibodies based on EVs	• High purity and selectivity	• High-cost antibodies	Momen-Heravi et al. (2013); Kang et al. (2017)
			• Elution may damage native EV structure	
Polymer-based precipitation	Polymers reduce EVs solubility by creating a hydrophobic microenvironment	• Easy operation	• Low purity	Niu et al. (2017); Moon et al. (2019)
		• High yield	• Polymers affect downstream MS analysis	

**FIGURE 1:**
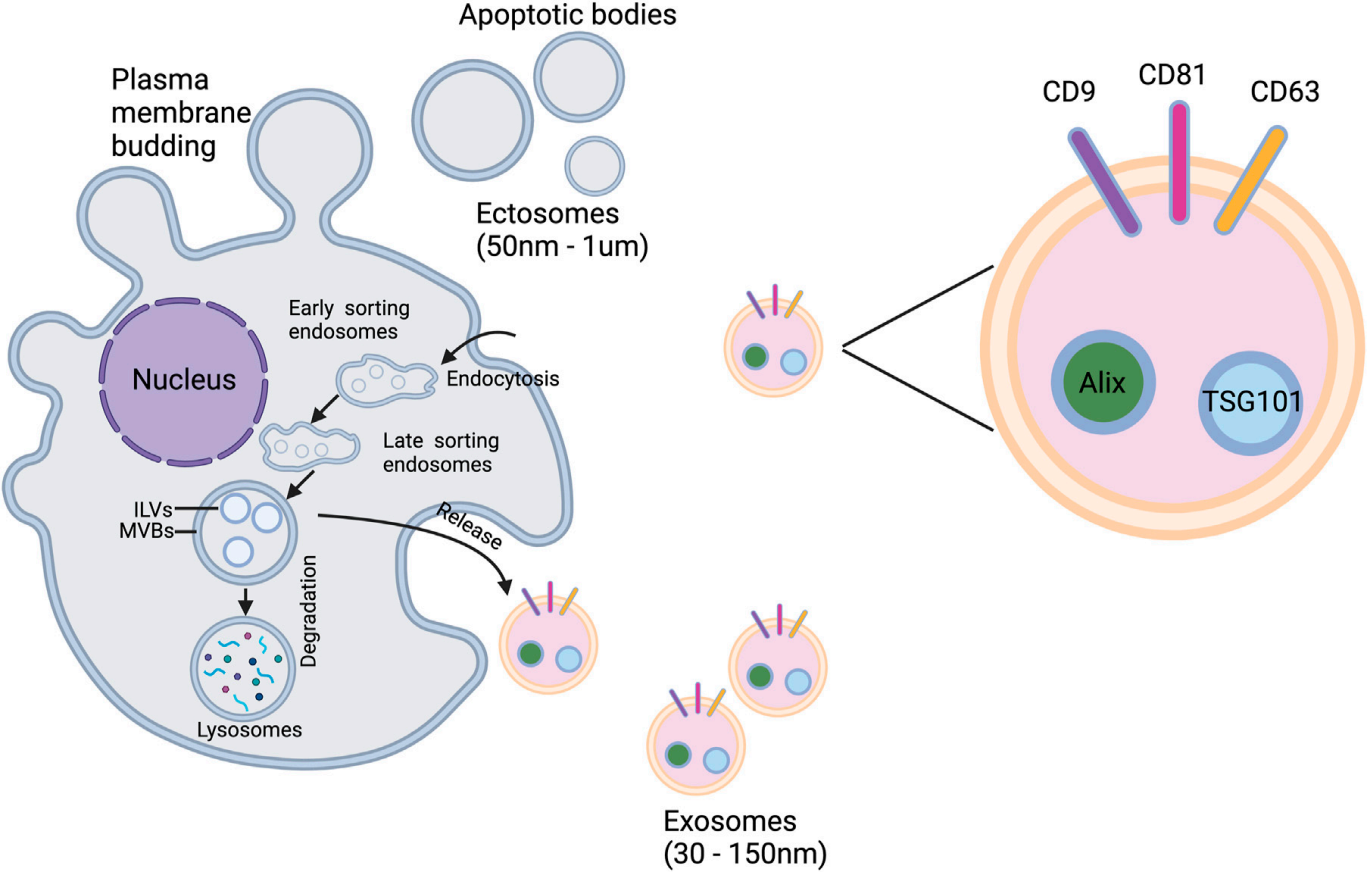
Two major types of EVs formation and release methods.

**FIGURE 2:**
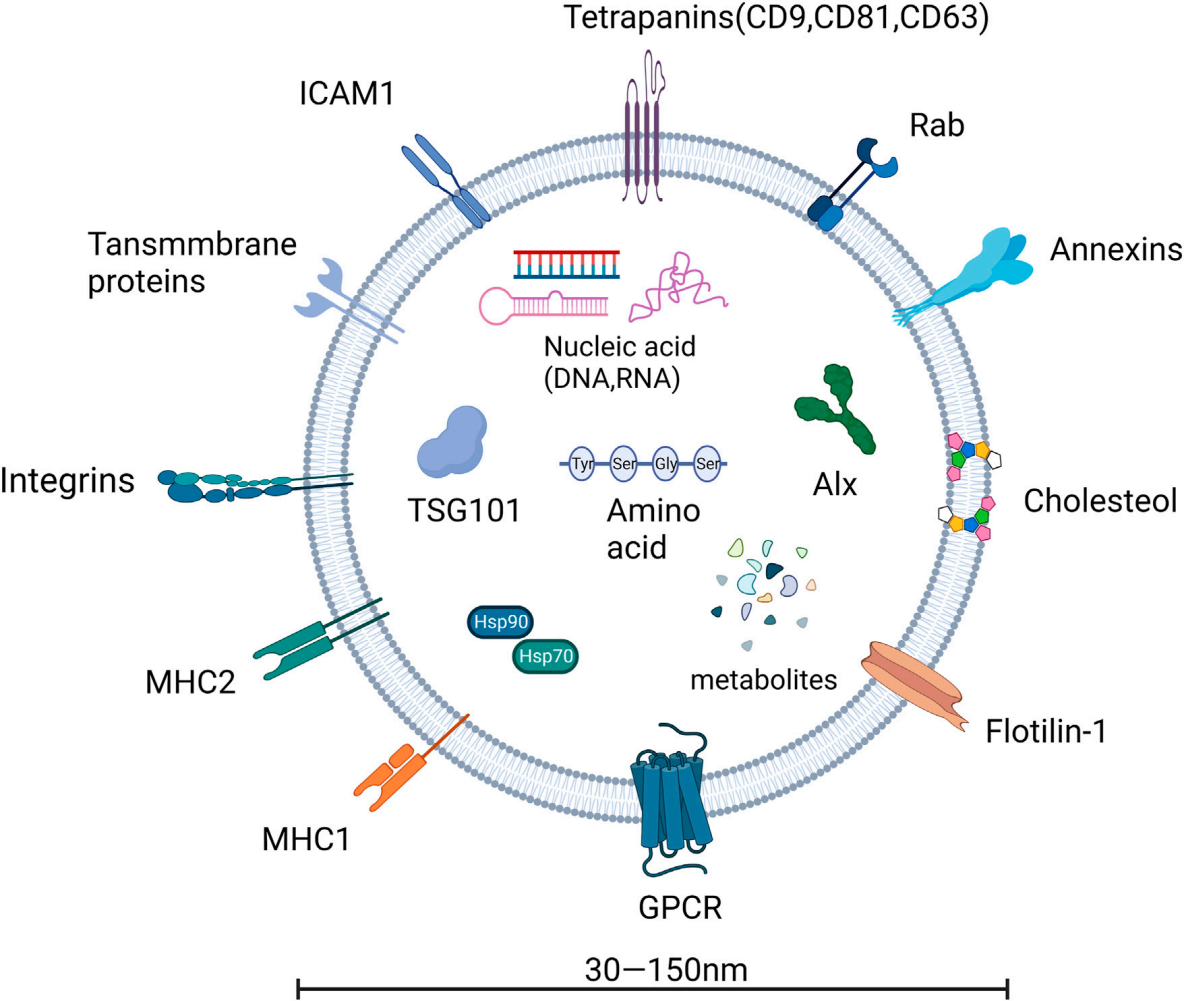
The basic structure of exosomes.

EVs are derived from the endosomal system and form intraluminal vesicles (ILVs) in multivesicular bodies (MVBs). This ILVs are used to degrade, recycle, or exocytose proteins, lipids and nucleic acids. Endosomes are classified into three types in the endosomal system or endocytic pathway: early endosomes, late endosomes, and circulating endosomes (Grant and Donaldson, 2009). Endosomes are formed by invagination of the plasma membrane. When the contents are destined to be degraded, recycled, or secreted, early endosomes can fuse with endocytic vesicles. The recycled content is partitioned into recycled endosomes (Morelli et al., 2004), and the remaining early endosomes are transformed into late endosomes (Stoorvogel et al., 1991). Late endosomes accumulate ILVs formed by endosomal membrane inward outgrowth. Cellular proteins, nucleic acids, and lipids are sorted into these vesicles during this process, and late endosomes containing numerous small vesicles are known as MVBs, which can fuse with lysosomes or the cell membrane, releasing ILVs into the extracellular space as exosomes (Grant and Donaldson, 2009), as shown in Figures 1, 2.

### Applications of Mφs-EVs

Many therapeutic NPs platforms, such as liposomes, albumin NPs, and polymeric micelles, have been approved for cancer treatment, and many other nanotechnology-enabled therapeutic modalities, such as chemotherapy, thermotherapy, radiation therapy, gene or RNA interference (RNAi) therapy, and immunotherapy, are playing an important role in the clinic. The complexity and heterogeneity of tumor biology, an incomplete understanding of nano-biological interactions, and the chemical, manufacturing, and control challenges required for clinical translation and commercialization are the major barriers to nanomedicine becoming a new paradigm for cancer treatment. Indeed, the rising mortality rate, a lack of treatments, and some of their side effects all contribute to the search for a more effective therapy, which nanomedicine is well suited for (Shi et al., 2017). Because of their important roles in NP-TME interactions, the applications of Mφs-EVs as an emerging nanomedicine technology are widely recognized as a promising therapeutic modality (Daldrup-Link et al., 2011; Miller et al., 2015) Figure 3.

**FIGURE 3:**
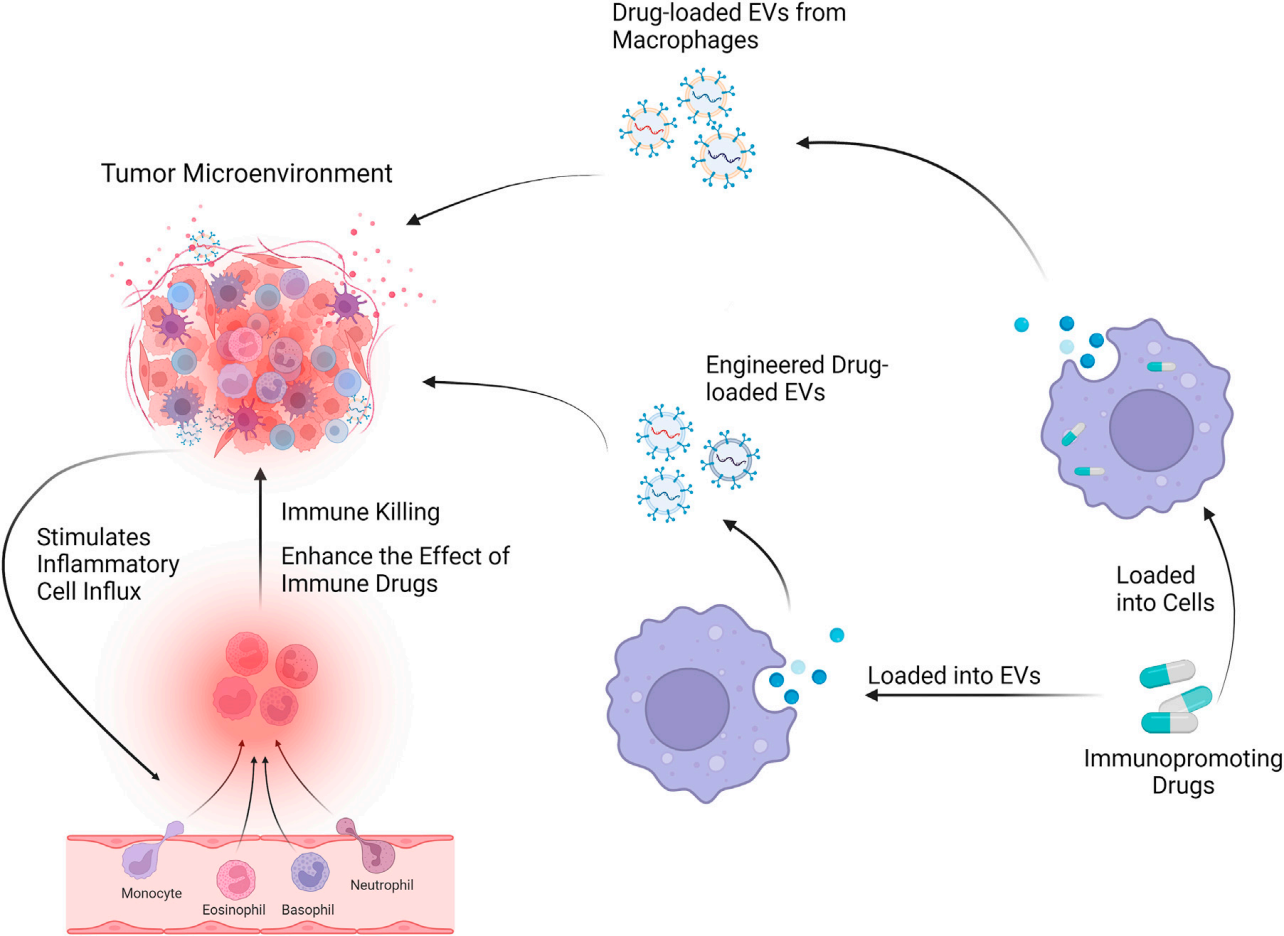
Mφs-EVs can be loaded with drugs and will affect the tumor microenvironment through drug delivery systems and engineered EVs.

### Drug delivery and tumor microenvironment

#### Drug delivery systems for Mφs-EVs

By incorporating leukocyte plasma membrane proteins into lipid NPs, it has been demonstrated that the resulting innovative hybrid NPs have comparable physicochemical properties to conventional liposomes and are more biocompatible, with a significant increase in affinity for inflammatory vessels (Molinaro et al., 2016). While Mφs have a higher affinity for tumor and inflammatory tissues than leukocytes, it has been demonstrated that these immune cells have a high affinity for these pathological environments due to the interaction of surface proteins with adhesion molecules, thus improving the targeting of the resulting nanosystems to TME (Belli et al., 2018; Molinaro et al., 2020). In besides, because Mφs-EVs have the inherent ability to cross natural barriers *in vivo*, they can specifically deliver payloads to hard-to-reach sites such as the central nervous system and tumor sites while minimizing side effects on healthy tissues (Yuan et al., 2017; Haney et al., 2020). Based on these benefits, Mφs drug delivery approaches are gradually gaining traction.

There is evidence that loading drugs into Mφs-EVs *via* the parent cell results in the formation of EVs-drug-loaded Mφs, and then EVs produced by EVs-drug-loaded Mφs are applied to cancer cells or tissues, and EVs produced by EVs-drug-loaded Mφs exhibit higher targeting to cancer cells, are more resistant in treating drug-resistant cancers with more significant potential and reduced tumor metastasis and showed higher anticancer efficacy in mouse tumor models. Thus, it is evident that Mφs-based EVs could provide a novel solution to a currently unmet clinical need while also reducing morbidity and mortality in cancer patients (Kim et al., 2016; Haney et al., 2020).

#### Applications of M1-Mφs-EVs formulations in tumors

Furthermore, when compared to M2, M1-derived EVs could create a pro-inflammatory microenvironment that increased the efficacy of chemotherapeutic drugs, whereas drug-loaded EVs had better anticancer effects (Wang et al., 2019). Exosome-mimetic nanovesicles derived from M1 (M1NVs) injections have been shown to repolarize M2 TAM into M1 and improve the antitumor efficacy of checkpoint inhibitor therapy (Choo et al., 2018). Two distinct drug delivery methods emerged as a result of this: 1) specific drug delivery systems based on M1 Mφs derived extracellular vesicles (M1EVs); 2) specific drug delivery systems based on modulated M1-EVs (MM1EVs). The first method directly loads a single drug into M1 Mφs, increasing the drug’s chemosensitivity. And this nanoformulation can significantly improve the efficiency on tumors by inhibiting cancer cell proliferation, cell attachment and migration, and chemoresistance to drugs (Zhao et al., 2021). The second method, involved loading multiple drugs onto MM1EVs, resulting in a synergistic effect of multiple drugs. The M1EVs are first functionalized with some drugs to form MM1EVs, and the remaining synergistic drugs are then loaded into the MM1EVs. These loaded nanosystems can cross the blood-brain barrier, accumulating in the TME and stimulating *in situ* repolarization of M2 to M1 TAMs. Immunomodulation and other pharmacological chemotherapy were combined to produce a powerful therapeutic effect. Furthermore, it was tested successfully on 3D *in vitro* and *in vivo* models, demonstrating superior anticancer effects and a safe profile on healthy tissues in both cell-derived and patient-derived xenograft models (Jorquera-Cordero et al., 2022; Wang et al., 2022).

**FIGURE 4:**
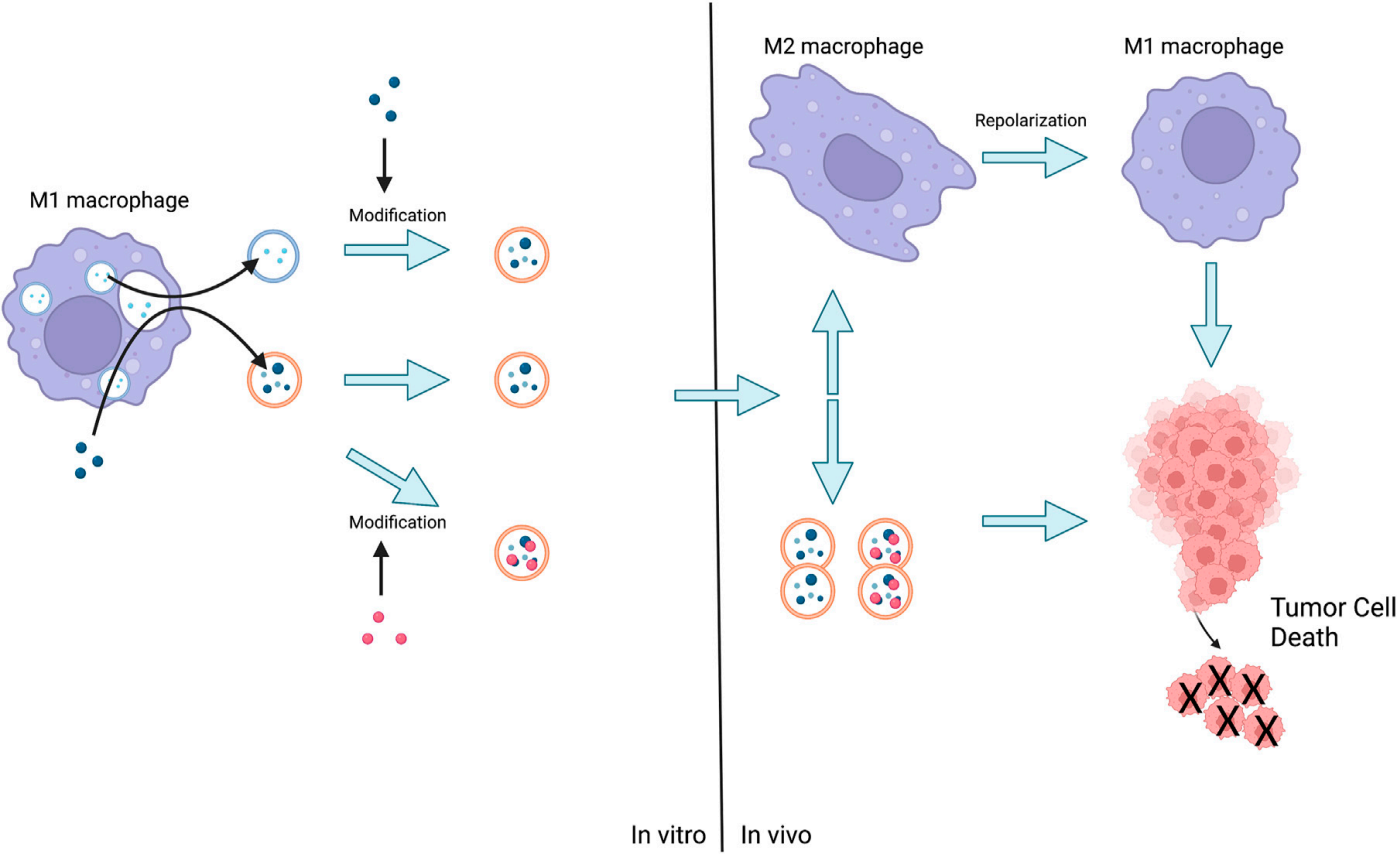
The method of action of Mφs-EVs is either the release of EVs by M1 followed by the administration of the loaded drug, or the stimulation of M1 by the advance administration of the drug and the subsequent production of EVs for direct action or further modification. Eventually EVs act on tumor cells or act on M2 leading to their repolarization and then act on tumor cells leading to their death.

As previously stated, the surface structure and cargo of EVs are similar to those of parental progenitor cells, and for this reason, Mφs-EVs hold great promise for applications that can bypass the body’s defense system, maintain somatic circulation for long periods, and provide tumor suppressive functions with the same targeting and intrinsic properties. Indeed, loading EVs isolated from will drugs with Mφs could be a promising way to improve cancer treatment efficacy Figure 4.

#### Vaccines for tumors

Tumor immunotherapy’s function is primarily based on the patient’s own immune system, and it is regarded as a significant shift in tumor treatment. Tumor vaccines, on the other hand, introduce tumor antigens into patients *via* a variety of pathways to overcome the immunosuppressive state caused by tumors, improve immunogenicity, activate the patient’s own immune system, and induce cellular and humoral immune responses in the body. Tumor vaccines, on the other hand, are ineffective on their own and must usually be used in conjunction with other therapies or adjuvants to improve the extent, breadth, quality, and longevity of the vaccine immune response (Finn, 2008). Indeed, certain factors released by Mφs themselves (e.g., insulin-like growth factor, IGF-1) can play an important role in vaccine-induced immune responses by regulating immune cell homeostasis (Yoon et al., 2017). Vaccines formulated with EVs, on the other hand, are very effective adjuvants that can increase the release of pro-inflammatory cytokines to induce better cross-activation of T cells and to inhibit tumor metastasis (Shi et al., 2020).

Mφs-sEVs have been shown to play a role in disease prevention. sEVs secreted by Mφs can downregulate pro-inflammatory miRNA target genes in receptor cells when stimulated with lipopolysaccharide (LPS) (Jean-Toussaint et al., 2021). In animal models, prophylactic intrathecal injection of sEVs facilitated chronic pain attenuation while having no effect on the response to protective physiological or acute inflammatory pain (McDonald et al., 2014). LPS stimulation altered the transcriptome of Mφs-sEVs in addition to miRNA, and transcriptome sequencing of RNA from sEVs revealed significant changes in innate and adaptive immune pathways, including those associated with NF-B activation and the TLR cascade.

Furthermore, Mφs-derived implantable vaccines can cause a switch from anti-inflammatory M2 Mφs to pro-inflammatory M1 Mφs. Furthermore, these vaccines can stimulate systemic immunity by promoting the maturation of dendritic cells (DCs) and the activation of memory T (TEM) cells in the tumor microenvironment, resulting in a self-replicating cycle with a more robust immune response (Wang et al., 2021c). Cheng et al. (Cheng et al., 2017) found an increased proportion of pro-inflammatory cytokines in an animal model of melanoma after combining a peptide vaccine with M1EVs administered subcutaneously 4 h before the peptide vaccine reached the tumor. When compared to conventional adjuvants such as TLR agonists, this combination induced a more intense and longer-lasting immune response and had a stronger anti-tumor effect.

M1EVs may act as an agent that stimulates the pro-inflammatory environment and increases the immune system’s sensitivity to cancer vaccines, resulting in superior anti-tumor effects. These findings strongly elaborat that M1EVs are a promising immune adjuvant that could be used in cancer vaccines.

#### Engineering Mφs-EVs in cancers

While targeted drug delivery can increase the local concentration and tumor targeting of therapeutic agents and minimize side effects, engineered EVs enable targeted drug delivery *via* genetic and chemical methods with the advantages of low toxicity, low immunogenicity, and high engineerability (Liang et al., 2021). Several technical approaches have been investigated (Armstrong et al., 2017; Ye et al., 2018; Cheng et al., 2021; Evers et al., 2022), and the main strategies are surface functionalization of EVs with various targeting molecules to enhance their specificity for cancer cells and/or hybridization with traditional synthetic nanosystems (e.g., liposomes) to provide specific physicochemical properties to the resulting hybrid vesicles. The therapeutic application of EVs may be limited by unwanted off-target activity and potential “dilution effects” during systemic administration, which may impair their ability of reaching target tissues, although preliminary clinical trials are currently underway.

#### Applications of engineered EVs

Gregor et al. (Fuhrmann et al., 2018) developed an EVs-based hydrogel for topical and controlled anti-inflammatory drug delivery. Hydrogels are useful for controlling drug release and simulating biomechanical functions (Zhang and Khademhosseini, 2017). Encapsulating EVs in biocompatible PVA hydrogels is a promising approach not only for enzyme prodrug therapy (EPT) as well as other therapeutic routes. Furthermore, the simple method of incorporating EVs into hydrogels could be extended to other biological applications such as local delivery of EVs or tissue engineering using EVs-containing scaffolds, thereby amplifying the EVs approach. Li et al. (Li et al., 2022) used platelet membrane-engineered EVs for targeted immunomodulatory therapy of cardiac repair. Intravenously injected platelet membrane-modified EVs (P-EVs) were carried into the ischemic myocardium primarily by circulating monocytes in a mouse model of MI/R injury. These monocytes then differentiate into Mφs in the inflammatory microenvironment, and the differentiated Mφs endocytose superficial P-EVs in large numbers *in situ*. P-EVs then successfully escape from Mφs lysosomes and release functional microRNAs (miRNAs) into the cell membrane, allowing M1 Mφs to be reprogrammed into M2 Mφs. Eventually, the immune microenvironment is controlled to allow for cardiac repair. Thus, monocytes mediated the migration of P-EVs into the ischemic myocardium, and P-EVs were endocytosed *in situ* by monocyte-derived Mφs, implying that monocyte-derived Mφs may have future immunomodulation potential in MI/R and other immune-related diseases. Although the above approach has not yet been used in Mφs-EVs, it does provide us with novel ideas for future engineering treatments for Mφs-EVs.

#### Applications of engineered Mφ-EVs

Indeed, with extensive research on Mφs-EVs, engineering of Mφs-EVs for therapeutic use has become increasingly abundant. Kim et al. (Kim et al., 2016) conjugated Mφs-EVs with the potent anticancer agent paclitaxel (PTX), resulting in a novel nanoformulation (EVs-PTX) with high anticancer efficacy in a mouse lung metastasis model. Kim et al. (Kim et al., 2018) improved on this novel nanoformulation by including aminoacetamide-polyethylene glycol (AA-PEG) carrier molecules to target the sigma receptors overexpressed by lung cancer cells. PTX-loaded AA-PEG carrier exosomes (AA-PEG-EVs-PTX) have a high loading capacity, accumulate strongly in cancer cells following systemic administration, and improved therapeutic efficacy. The formulation has excellent structural and therapeutic indices for systemic administration, making it a powerful and novel anticancer delivery platform.

Li et al. (Li et al., 2020) developed an Mφs-EVs-encapsulated (lactic-ethanolic acid) poly-nanoplatform for targeted chemotherapy of Triple-negative breast cancer (TNBC). A peptide was added to the surface of the EVs to target the mesenchymal-epithelial transition factor (c-Met), which is overexpressed in TNBC cells. The results showed that the nanocarriers significantly improved cellular uptake efficiency, significantly increased tumor targeting efficacy, enhanced inhibition of tumor growth, and caused apoptosis. These results suggest that engineered NPs encapsulated with Mφs-EVs are a promising drug delivery strategy for TNBC therapy. Following that, Gong et al. (Gong et al., 2019) demonstrated EV functionalization by stimulating THP-1 cells with 12-myristate 13-acetate (PMA), which resulted in the overexpression of catabolic proteins and metalloproteinase 15 (A15) on the surface of derived EVs. A15 contains an RGD motif that enhances interaction with v3 integrins and may be involved in cell migration at multiple levels (Martin et al., 2002; Zhong et al., 2008; Danhier et al., 2012). This allowed therapeutic amounts of doxorubicin (Dox) and cholesterol-modified miRNA 159 (Cho-miR159) to be delivered to TNBC cells *in vitro* and *in vivo*. Protein kinase C continuously activated targeted A15-EVs in Mφs. These cell-derived EVs were targeted and produced 2.97-fold more. In MDA-MB-231 cells, A15-EVs co-loaded with Dox and Cho-miR159 induced a synergistic therapeutic effect. *In vitro*, A15-EVs co-loaded with Dox and Cho-miR159 induced a synergistic therapeutic effect in MDA-MB-231 cells. *In vivo*, vesicular delivery of miR159 and Dox effectively silenced the TCF-7 gene and demonstrated improved anticancer effects with no side effects. Li et al. (Li et al., 2021) encapsulated tumor-targeting folate (FA)-modified EVs with intracellularly produced protoporphyrin X (PpIX) and doxorubicin (DOX), and demonstrated that, when compared to conventional EVs with direct drug infusion or drug infusion, these biosynthetic EVs demonstrated higher drug loading efficiency, minimized structural and functional perturbations, and enhanced immune response, ablating orthotopic and meta.

An alternative to surface modification and loading procedures directly for the post-modification of EVs is to implement hybrid nanosystems made up of EVs and conventional nanocarriers to maximize the benefits of both delivery systems (Lv et al., 2020). By synthesizing genetically engineered exosome-thermo-sensitive liposome hybrid NPs, Lv et al. (Lv et al., 2020) discovered that hybrid NPs could efficiently penetrate mPC tumors after intravenous injection and release payload under the hypothermic conditions of HIPEC by synthesizing genetically engineered exosome-thermo-sensitive liposome hybrid NPs, overcoming the barrier of low drug delivery efficiency to some extent. Rayamajhi et al. (Rayamajhi et al., 2019) created hybrid exosomes (HE) with sEVs and synthetic liposomes that were less than 200 nm in size. The HE was then loaded with DOX. The drug-loaded HE demonstrated enhanced cancer cell toxicity and pH-sensitive drug release under acidic conditions, facilitating drug delivery in the acidic cancer environment. These findings imply that engineered HE could be a promising platform for tumor-targeted drug delivery.

All of the strategies described here demonstrate the design of Mφs-EVs using various technical approaches to improve their potential application in cancer therapy. As expected, these NPs can be well adapted to a variety of modification processes, demonstrating great versatility. We believe that the modification of the parent cell to induce specific features of the released EVs, as well as the hybridization of EVs with conventional drug delivery systems, is a promising therapeutic approach among the various pathways investigated thus far. This latest nanomedicine approach, in particular, has the potential to greatly improve the characteristics of the original EVs, not only by utilizing the natural properties of EVs, but also by displaying the specific physicochemical properties provided by synthetic nanocarriers.

## Discussion and prospects

Cancer affects people of all ages all over the world, but no effective cure is commercially available. In this context, scientists have begun to investigate all possible approaches to achieving a safe and effective treatment. Nanotechnology and biology have recently produced promising results in the field of EVs. EVs are a type of communication mediator that enters the body and delivers bioactive substances such as lipids, RNA, DNA, and proteins. They participate in numerous physiological and pathological pathways. Mφs play an important role in TME, and Mφs-EVs function similarly as a subclass of EVs, exhibiting typical macrophagic characteristics such as tropism towards inflammatory tissues and tumor sites. M1 Mφs are expected to be the source of EVs among the Mφs phenotypes, and their derived EVs have a significant ability to inhibit or promote inflammation, which will aid in achieving a personalized and effective anti-cancer response. Simultaneously, with appropriate modifications, these nano-vesicles may provide a suitable drug-targeting nano-platform, laying the groundwork for the development of “next-generation” anticancer nanomedicines.

The role of Mφs in cancer suppression or progression opens the possibility of manipulating Mφs *via* metabolic reprogramming to create novel therapeutic approaches. Mφs metabolism, on the other hand, is increasingly recognized as a complex, tightly regulated phenomenon that affects and/or is influenced by various tumor cell and TME characteristics. Future research and therapeutic efforts focusing on Mφs metabolism appear insufficient without taking into account the complex regulation of Mφs metabolism by internal and external factors. Furthermore, the difficult processing, complicated process, and high cost of traditional Mφs-EV extraction methods, as well as the immaturity of tumor-related nanotechnology, severely limit their future biomedical applications. In conclusion, we need a deeper understanding of the properties and potential value of Mφs derived exosomes to meet the challenges posed by rapidly evolving biology and cancer nanotechnology.
